# Elucidating a normal function of huntingtin by functional and microarray analysis of huntingtin-null mouse embryonic fibroblasts

**DOI:** 10.1186/1471-2202-9-38

**Published:** 2008-04-15

**Authors:** Hua Zhang, Sudipto Das, Quan-Zhen Li, Ioannis Dragatsis, Joyce Repa, Scott Zeitlin, György Hajnóczky, Ilya Bezprozvanny

**Affiliations:** 1 Department of Physiology, UT Southwestern Medical Center at Dallas, Dallas, TX 75390, USA; 2 Department of Pathology and Cell Biology, Thomas Jefferson University, Philadelphia, PA 19107, USA; 3 Department of Immunology, UT Southwestern Medical Center at Dallas, Dallas, TX 75390, USA; 4 Department of Physiology, The University of Tennessee Health Science Center, Memphis, TN 38163, USA; 5 Department of Neuroscience, University of Virginia School of Medicine, Charlottesville, VA 22908, USA

## Abstract

**Background:**

The polyglutamine expansion in huntingtin (Htt) protein is a cause of Huntington's disease (HD). Htt is an essential gene as deletion of the mouse Htt gene homolog (*Hdh*) is embryonic lethal in mice. Therefore, in addition to elucidating the mechanisms responsible for polyQ-mediated pathology, it is also important to understand the normal function of Htt protein for both basic biology and for HD.

**Results:**

To systematically search for a mouse Htt function, we took advantage of the *Hdh *+/- and *Hdh*-floxed mice and generated four mouse embryonic fibroblast (MEF) cells lines which contain a single copy of the *Hdh *gene (*Hdh*-HET) and four MEF lines in which the *Hdh *gene was deleted (*Hdh*-KO). The function of Htt in calcium (Ca^2+^) signaling was analyzed in Ca^2+ ^imaging experiments with generated cell lines. We found that the cytoplasmic Ca^2+ ^spikes resulting from the activation of inositol 1,4,5-trisphosphate receptor (InsP_3_R) and the ensuing mitochondrial Ca^2+ ^signals were suppressed in the *Hdh*-KO cells when compared to *Hdh*-HET cells. Furthermore, in experiments with permeabilized cells we found that the InsP_3_-sensitivity of Ca^2+ ^mobilization from endoplasmic reticulum was reduced in *Hdh*-KO cells. These results indicated that Htt plays an important role in modulating InsP_3_R-mediated Ca^2+ ^signaling. To further evaluate function of Htt, we performed genome-wide transcription profiling of generated *Hdh*-HET and *Hdh*-KO cells by microarray. Our results revealed that 106 unique transcripts were downregulated by more than two-fold with p < 0.05 and 173 unique transcripts were upregulated at least two-fold with p < 0.05 in *Hdh*-KO cells when compared to *Hdh*-HET cells. The microarray results were confirmed by quantitative real-time PCR for a number of affected transcripts. Several signaling pathways affected by *Hdh *gene deletion were identified from annotation of the microarray results.

**Conclusion:**

Functional analysis of generated Htt-null MEF cells revealed that Htt plays a direct role in Ca^2+ ^signaling by modulating InsP_3_R sensitivity to InsP_3_. The genome-wide transcriptional profiling of Htt-null cells yielded novel and unique information about the normal function of Htt in cells, which may contribute to our understanding and treatment of HD.

## Background

Huntington's disease (HD) is an autosomal-dominant neurodegenerative disorder which is caused by polyglutamine (polyQ) expansion in the amino-terminus of huntingtin (Htt). Htt is a soluble protein of 3,144 amino acids that has no sequence homology with other proteins. Except for the extreme amino-terminus, with its adjacent polyQ region and proline-rich segments, the entire ~350-kD protein is predicted to be composed of 36 α-helical HEAT repeats. Increasing evidence indicates that Htt functions as a molecular scaffold that is able to organize a variety of signaling complexes [1,2]. Htt is expressed ubiquitously in humans and rodents, with the highest levels found in CNS neurons and the testes [3-5]. Intracellularly, Htt is associated with various organelles, including the nucleus, endoplasmic reticulum (ER) and Golgi complex [6-8]. This widespread subcellular localization does not facilitate the definition of its function. Hdh is evolutionary conserved – a single copy of the Htt gene is expressed in all vertebrates (from fish to humans) [9]. The Htt gene is also present in *D. melanogaster *genome, but absent in the *C. elegans *and *S. cerevisiae *genomes [9]. All vertebrate isoforms of Htt, but not *Drosophila *Htt, contain an amino-terminal polyQ region.

Complete knockout of the mouse Htt gene (*Hdh*) causes embryonic death before day 8.5 (E8.5, before gastrulation and the formation of the nervous system) [10-12]. After gastrulation, Htt becomes important for neurogenesis – mice carrying a <50% dose of wild-type Htt display profound malformations of the cortex and striatum [13]. Another study has shown that greatly reduced Htt levels are insufficient to support normal mouse development [14]. In addition to its function in development, Htt may play a role in the regulation of apoptosis, control of BDNF production, vesicular and mitochondrial transport, neuronal gene transcription, and synaptic transmission (reviewed in [9]). Despite all of these efforts and results, the exact function of Htt in cells still remains largely unknown.

In addition to answering an academic question concerning the normal function of Htt, knowledge of its function is important for understanding HD pathogenesis and for the treatment of Huntington's disease (HD). Although the HD mutation is considered to be a "gain of function" mutation, it has been suggested that the loss of normal Htt function might also contribute to the pathogenesis of HD [9]. Approaches that are based on reducing mutant Htt expression such as RNA interference [15] and the use of intrabodies [16,17] are currently considered to be promising strategies for HD treatment. It is likely that these agents will cause inactivation or impair normal function of both mutant and wild type Htt alleles. One can envision a therapy that combines such Htt-inactivating agents with drugs that restore the function of targets and pathways downstream from wild-type Htt. However, because both normal Hdh function is not known and downstream pathways have not been identified, such a combined therapy approach is not feasible at the moment.

To systematically search for Htt's normal function, we used *Hdh *+/- [12] and *Hdh*-floxed mice [18] to generate immortalized mouse embryonic fibroblasts (MEF) which contain a single functional copy of *Hdh *gene (*Hdh*-HET) or lack *Hdh *completely (*Hdh*-KO). We compared inositol 1,4,5-trisphosphate receptor (InsP_3_R)-mediated Ca^2+ ^signals in these cells. We then performed a genome-wide gene transcription profiling of *Hdh*-HET and *Hdh*-KO MEF cells using microarrays to obtain novel, unique, and unbiased information about the normal function of Htt in fibroblasts, which may contribute to our understanding and treatment of HD.

## Results

### Generation of *Hdh*-HET and *Hdh*-KO MEF cell lines

**Figure 1:**
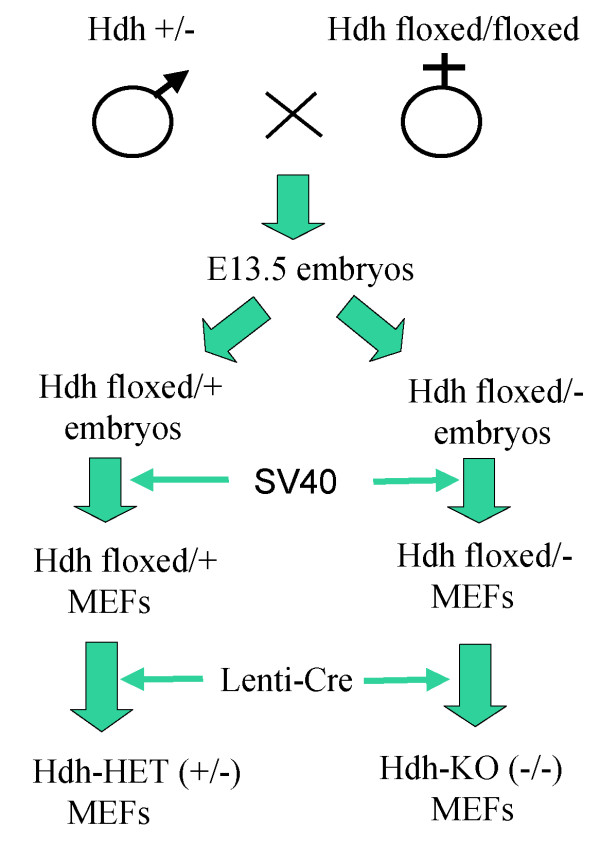
**Experimental procedure used to generate the *Hdh*-HET and *Hdh*-KO MEF cell lines**. The flow chart schematically describes the experimental paradigm used to generate *Hdh*-HET and *Hdh*-KO MEF cell lines. Please see text for details.

**Figure 2:**
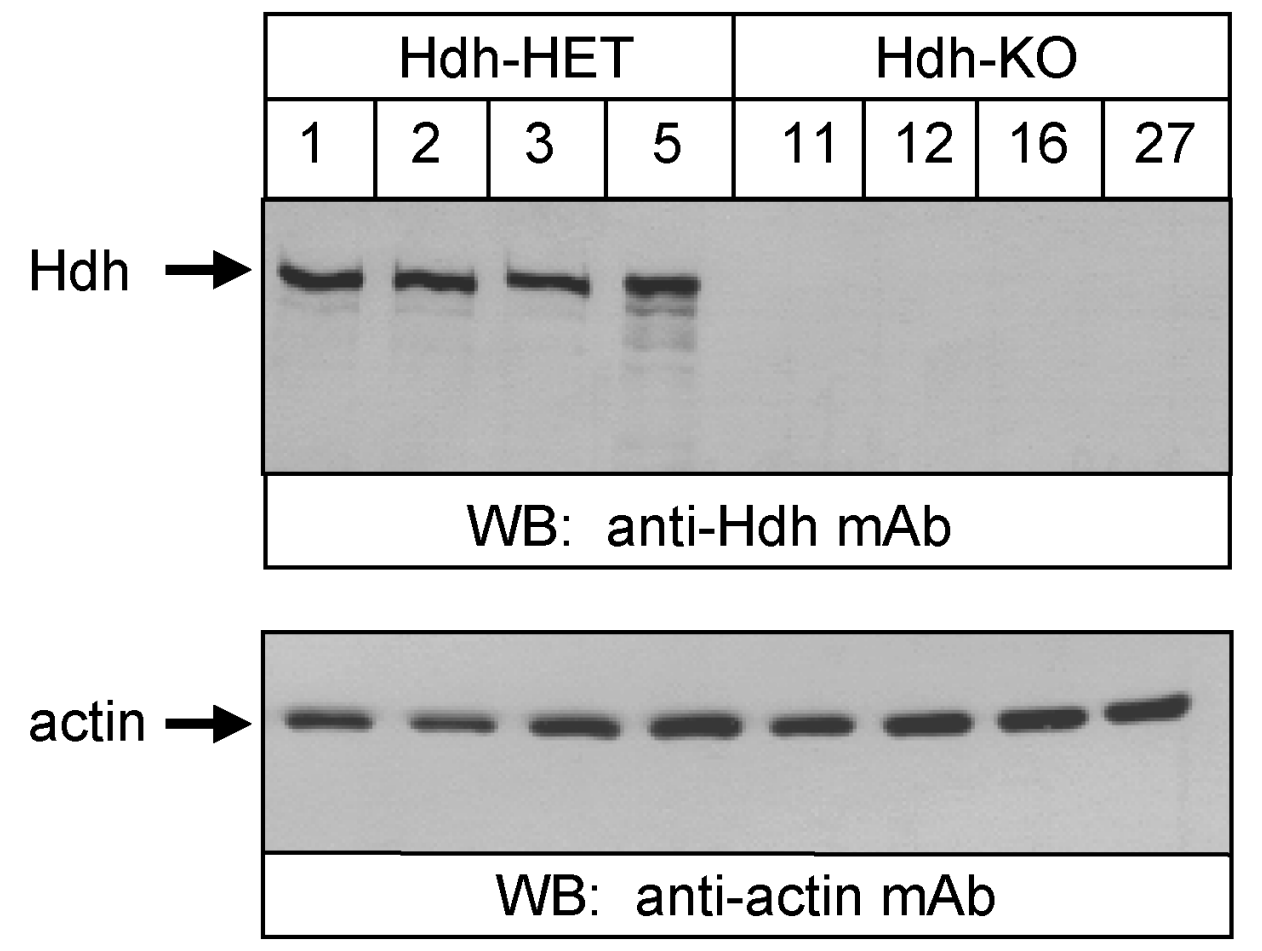
**Western blotting analysis of *Hdh*-HET and *Hdh*-KO MEF lines**. The whole cell lysates from 4 *Hdh*-HET and 4 *Hdh*-KO MEF cel lines were probed with anti-Htt monoclonal antibody. The same samples were also probed with anti-actin monoclonal antibody as a loading control. The ECL was used for detection.

To generate cell lines lacking Htt expression, we employed a conditional mutagenesis strategy based on the *in vitro *recombination of an *Hdh*(flox) allele in cultured fibroblasts that are also carrying either a wild-type (+) or null (standard knock-out) *Hdh *allele. The *Hdh*(flox/+) and *Hdh*(flox/-) fibroblasts were obtained from embryos derived from a cross between *Hdh *+/- and *Hdh*-floxed/floxed mice (Fig 1). Primary fibroblasts were prepared and plated separately from each embryo as described in Methods. After two days in culture, the primary fibroblasts from all embryos with identical genotype (*Hdh *floxed/+ or *Hdh *floxed/-) were pooled together and transfected with a linearized SV40 plasmid. Transfected cells were then cultured for four to six weeks until immortalized *Hdh *floxed/+ and *Hdh *floxed/- mouse embryonic fibroblasts (MEFs) were obtained (Fig 1). To recombine the *Hdh *floxed allele, immortalized *Hdh *floxed/+ and *Hdh *floxed/- MEFs were infected with Lenti-NLS-GFP-Cre virus encoding nuclear-targeted GFP-Cre fusion protein [19] (Fig 1). Using the procedure described above, we generated four *Hdh*-HET (lines 1,2,3,5) and four *Hdh*-KO (lines 11, 12, 16, 27) MEF cell lines. The expression of Htt in the generated MEF lines was assessed by Western blotting of whole cell lysates using anti-Htt monoclonal antibody. Quantification of Western blotting data verified similar levels of Htt expression in all 4 *Hdh*-HET lines (data not shown). Consistent with the genotype of the generated cells, we detected a protein of predicted size (~350 kD) in lysates from the *Hdh*-HET cells, but not in lysates from *Hdh*-KO lines (Fig 2). The same samples were probed with monoclonal antibodies against β-actin as a loading control (Fig 2). Thus, we concluded that we successfully generated four *Hdh*-HET and four *Hdh*-KO MEF lines on similar genetic background. We reasoned that comparison of resulting MEF lines may reveal clues about normal function of Htt protein in cells.

### Intracellular calcium signaling in *Hdh*-HET and *Hdh*-KO MEF cell lines

Previous studies have implicated impaired calcium signaling in the pathogenesis of HD [20-22]. The Htt directly binds to the inositol 1,4,5-trisposphate receptor (InsP_3_R), an intracellular Ca^2+ ^release channel [23,24]. The expression of mutated Htt has been shown to affect the InsP_3_R activity [23] and mitochondrial Ca^2+ ^signals and bioenergetics [25-30]. Since Htt and mutated Htt directly targets both ER and mitochondrial sites, it is possible that Htt may have some relevance for the physical and local Ca^2+ ^coupling between ER and mitochondria [31,32]. To test this idea and to determine a role played by Htt in intracellular Ca^2+ ^signaling, we performed a series of cytosolic and mitochondrial Ca^2+ ^imaging experiments with generated MEF lines. Two *Hdh*-HET (HET1 and HET 5) and two *Hdh*-KO MEF cell lines (KO12 and KO27) (Fig 2) were selected for Ca^2+ ^imaging studies.

**Figure 3:**
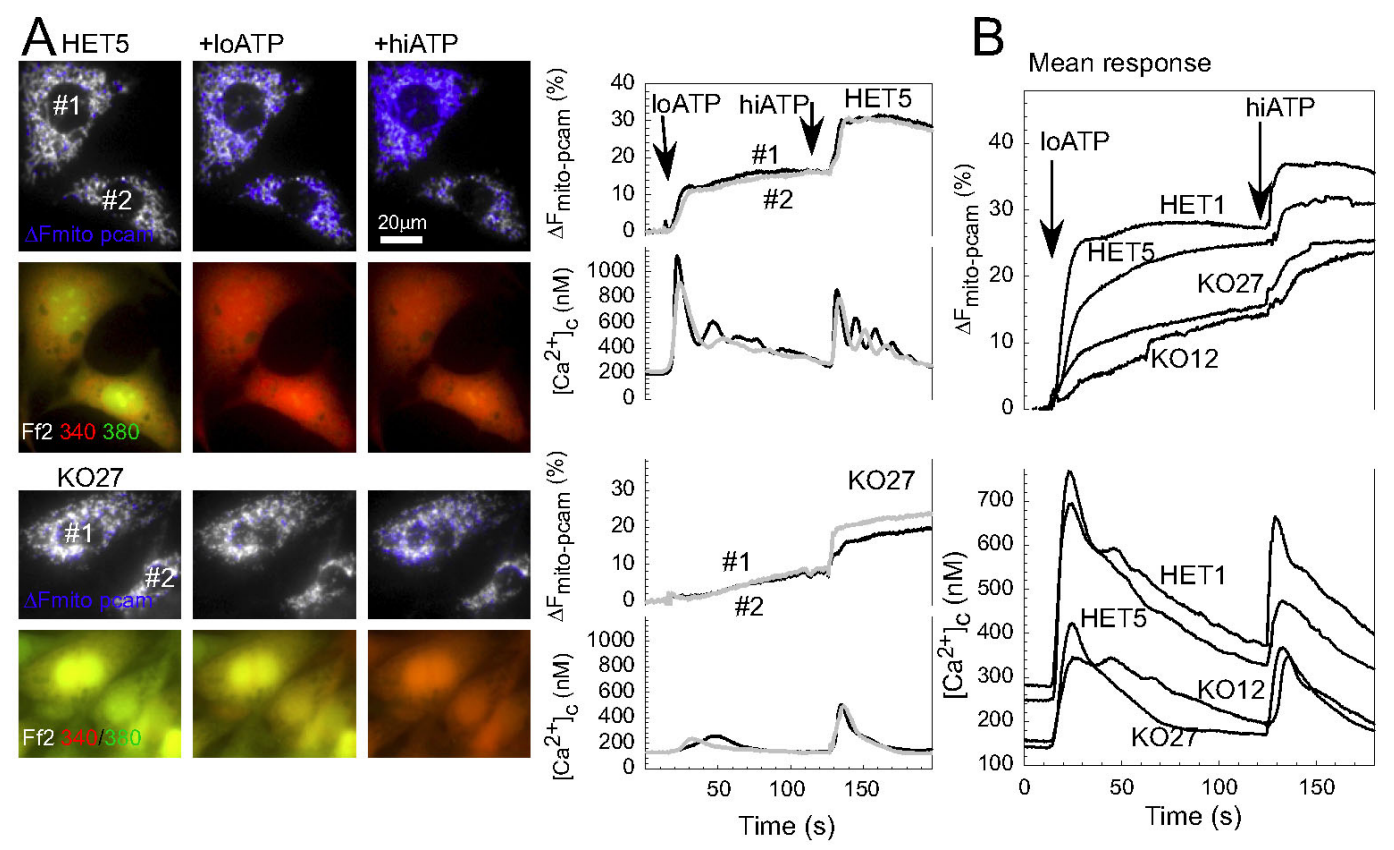
**Cytoplasmic and mitochondrial Ca^2+ ^signals in *Hdh*-HET and *Hdh*-KO cells**. MEF cells were transfected with mitochondrial matrix-targeted inverse pericam and were loaded with Fura-2/AM to monitor [Ca^2+^]_m _and [Ca^2+^]_c_, respectively. A. [Ca^2+^]_c _and [Ca^2+^]_m _signals in HET5 and KO27 cells. In the images, the inverse pericam fluorescence is shown in the gray scale; the blue overlay indicates the sites of the ATP-induced [Ca^2+^]_m _elevations (upper row of images). The Fura2 images are presented as green-red overlay where the [Ca^2+^]_c _elevations are indicated by a green to red shift (lower row). The graphs show the calibrated [Ca^2+^]_c _signal and the pericam fluorescence response (ΔF_mito-pcam_, decrease normalized to the initial fluorescence level) to the sequential stimulation by low (loATP, 2 μM) and high (hiATP, 100 μM) ATP in the single cells marked by the numbers. B. Mean [Ca^2+^]_c _and [Ca^2+^]_m _signals in *Hdh*-HET and *Hdh*-KO MEF cells. Traces show the mean of at least triplicate measurements for each cell line. The data are representative of four independent experiments.

In these experiments *Hdh*-HET and *Hdh*-KO MEF cell lines were challenged by ATP, an agonist of InsP_3 _signaling pathway in fibroblasts. Before stimulation with ATP the incubation medium was switched to a Ca^2+ ^free buffer to prevent Ca^2+ ^entry. The cytosolic [Ca^2+^]_c _and mitochondrial [Ca^2+^]_m _levels were monitored simultaneously as described in Methods. We found that pre-stimulation [Ca^2+^]_c _was higher in the *Hdh*-HET cells (HET1, 183 ± 11 nM (n = 42) and HET5 139 ± 9 nM (n = 33)) than that in the *Hdh*-KO cells (KO12, 102 ± 12 nM (n = 15) and KO27, 106 ± 6 nM (n = 48), p < 0.003). Stimulation with a suboptimal dose of ATP (2 μM) elicited large [Ca^2+^]_c _spikes in *Hdh*-HET cells, whereas only a small and slow [Ca^2+^]_c _rise was evoked in the *Hdh*-KO cells (Fig 3, Δ[Ca^2+^]_c_: HET1, 374 ± 24 nM; HET5, 386 ± 40 nM; KO12, 266 ± 47 nM and KO27, 284 ± 25 nM; p < 0.03). Many *Hdh*-KO cells did not show any [Ca^2+^]_c _rise in response to 2 μM ATP (not shown). Mobilization of the residual Ca^2+ ^by optimal ATP (100 μM) evoked a relatively large [Ca^2+^]_c _elevation in the *Hdh*-KO cells (Fig 3). The ATP-induced [Ca^2+^]_c _spikes were closely followed by a [Ca^2+^]_m _elevation in the *Hdh*-HET cells, whereas the [Ca^2+^]_m _rise was modest in the *Hdh*-KO cells (Fig 3). Thus, the ATP-induced intracellular Ca^2+ ^mobilization was suppressed and desensitized in the *Hdh*-KO cells.

Figure 3 has also showed that the [Ca^2+^]_c _signal was effectively propagated to the mitochondria in the *Hdh*-HET cells but less Ca^2+ ^was transferred into the mitochondria in the *Hdh*-KO cells. To determine whether this resulted from the attenuated ER Ca^2+ ^mobilization or from impaired ER-mitochondrial Ca^2+ ^coupling, the [Ca^2+^]_m _rise was plotted against the [Ca^2+^]_c _increase for each cell., The relationship between [Ca^2+^]_c _elevations and the ensuing [Ca^2+^]_m _signals was similar in both the *Hdh*-HET and in the *Hdh*-KO cells (not shown). This result indicated that the local coupling between ER and mitochondria is maintained in the *Hdh*-KO cells.

**Figure 4:**
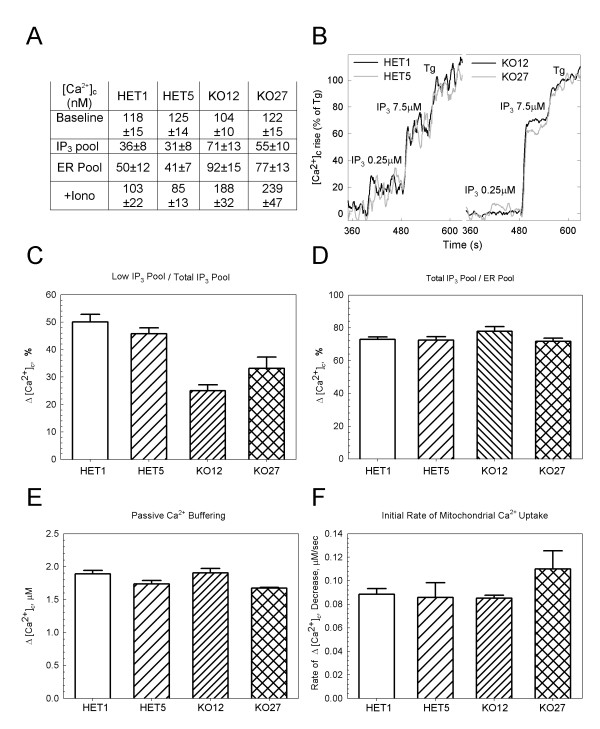
**Ca^2+ ^handling by *Hdh*-HET and *Hdh*-KO cells**. Fluorimetric measurements of cytoplasmic Ca^2+ ^using Fura-2 (A-D) or FuraFF (E and F) in permeabilized *Hdh*-HET and *Hdh*-KO MEF cells were conducted as described in *Materials and Methods*. A. Steady state [Ca^2+^]_c _values and the size of the intracellular calcium pools in HET1, HET5, KO12 and KO27 cells. Prestimulation steady state [Ca^2+^]_c _obtained in presence of ATP 2 mM, creatine phosphate 5 mM, creatine phosphokinase 5 units/ml and succinate 2 mM. To obtain the size of InsP_3_, ER and total ionomycin-sensitive pools, 7.5 μM InsP_3_, 2 μM thapsigargin (Tg) and 10 μM ionomycin were added respectively. Mean ± SE of at least five independent experiments with multiple parallels (HET1: n = 12; HET5; n = 12; KO12, n = 13; KO27, n = 10). B. Analog traces showing the [Ca^2+^]_c _responses to suboptimal (250 nM) and optimal InsP_3 _(7.5 μM) and Tg for HET1, HET5, KO12 and KO27 cells. Changes were normalized to the size of the ER Ca^2+ ^pool. C. Suboptimal InsP_3 _(250 nM)-induced [Ca^2+^]_c _increase normalized to total InsP_3_-sensitive (7.5 μM) pool. D. Maximal InsP_3 _(7.5 μM)-induced [Ca^2+^]_c _increase normalized to total Tg-sensitive (2 μM) ER pool. E. passive Ca^2+ ^buffering assessed as the [Ca^2+^]_c _increase evoked by the addition of 10 μM CaCl_2 _in Tg and Ruthenium Red (3 μM)- pretreated MEF cells. F. Mitochondrial Ca^2+ ^uptake caused by the addition of 40 μM CaCl_2_. Rate of uptake was calculated for initial 30 seconds after the challenge during which the rate was linear. Correction was made for the Ruthenium Red insensitive component. Data values show mean ± standard error of at least three independent experiments with multiple parallels. * indicates statistically significantly different p values (p < 0.03).

The attenuated ATP-induced [Ca^2+^]_c _signal in the *Hdh*-KO cells could be due to reduced InsP_3 _generation, due to reduced sensitivity of InsP_3_R or due to depleted ER Ca^2+ ^pool. To discriminate between these possibilities the InsP_3_-induced Ca^2+ ^mobilization was quantified in suspensions of permeabilized MEF cells. The steady state [Ca^2+^]_c _was similar in both *Hdh*-HET and in *Hdh*-KO cells (Fig 4A, HET1, n = 12; HET5, n = 12; KO12, n = 13; KO27, n = 10 measurements). The Ca^2+ ^pool size for both the ER and the ionophore-sensitive compartment was larger in the *Hdh*-KO than in the control cells (Fig 4A), whereas the uncoupler-sensitive mitochondrial compartment showed no difference (n = 3; not shown). Sequential application of a suboptimal and optimal InsP_3 _revealed lesser InsP_3_-sensitivity in the *Hdh*-KO cells than in the HET cells (Fig 4B, 4C, p < 0.01). However, the InsP_3 _sensitive fraction of the ER was approximately 75% in both control and *Hdh*-HET cells (Fig 4D). Furthermore, neither the passive Ca^2+ ^buffering nor the mitochondrial Ca^2+ ^uptake was altered in the cells lacking the *Hdh *(Fig 4E, 4F). Collectively, the data obtained in permeabilized cells suggest that the InsP_3 _sensitivity of the InsP_3 _receptor is attenuated in the *Hdh*-deficient cells, providing a mechanism to underlie the suppression of the InsP_3_-linked [Ca^2+^]_c _signaling in the *Hdh*-KO cells. The effect of *Hdh *on the InsP_3 _sensitivity is likely to be mediated by direct association between Htt and InsP_3_R [23,24].

### Microarray analysis of transcripts expressed in *Hdh*-KO and *Hdh*-HET MEFs

The results described in the previous section suggested that Htt plays a direct role in Ca^2+ ^signaling by modulating InsP_3_R function. Many studies suggested that Htt also plays a major role in control of gene transcription [33,34]. To uncover potential gene expression changes we performed genome-wide transcription profiling of *Hdh*-HET and *Hdh*-KO MEF cells. Using the procedures described in Methods, we isolated total RNA from *Hdh*-HET MEF lines 1, 2 (in duplicate), 3, and 5 (in duplicate) and from *Hdh*-KO MEF lines 11, 12, 16 (in duplicate), 27 (in duplicate). The resulting 12 samples were provided to the UT Southwestern Microarray Core Facility (MCF) for genome-wide expression profiling using Sentrix Mouse-6 Expression Bead Chips (Illumina) (see Methods for details).

**Figure 5:**
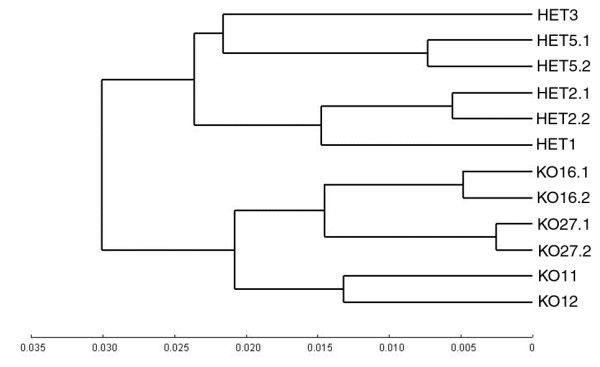
**Cluster analysis of microarray data**. Using the Illumina BeadStudio 1.5 software package, each of 12 arrays was treated as an independent experiment and cluster analysis was performed on the microarray hybridization results.

All 12 arrays produced highly reproducible and consistent gene expression data. The microarray results have been deposited in NCBIs Gene Expression Omnibus (GEO), and are accessible through GEO Series accession number GSE11139 [35]. Cluster analysis of the over-all gene expression data from the 12 samples demonstrated that all six *Hdh*-HET samples and all six *Hdh*-KO samples were clustered together, forming two clearly distinct groups (Fig 5). We also found that the duplicate samples (HET5.1 and HET5.2, HET2.1 and HET2.2, KO16.1 and KO16.2, and KO 27.1 and KO27.2) were most similar to each other when compared to other samples (Fig 5), as should be expected. Thus, we concluded that we obtained a high quality dataset of transcripts expressed in *Hdh*-HET and *Hdh*-KO MEF lines.

**Figure 6:**
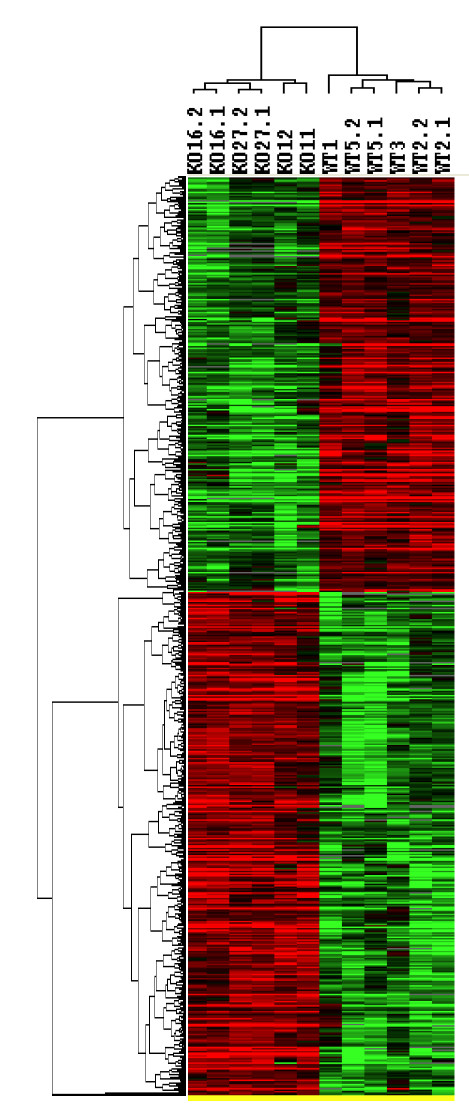
**Altered gene expression between *Hdh*-HET and *Hdh*-KO groups**. Cluster analysis of the 821 differentially expressed genes between *Hdh*-HET and *Hdh*-KO (p < 0.05). The genes were selected by comparing 6 HET samples with 6 KO samples using t-test with computing false discovery rate. The genes with p value <0.05 were selected and Hierarchical cluster analysis were performed using Cluster and TreeView . Each column represents a sample and each row represents a gene. The colorgram depicts high (red) and low (green) relative levels of gene expression in each sample.

In the next level of analysis, we combined all results obtained in six arrays with the *Hdh*-HET samples (HET group) and in six arrays with the *Hdh*-KO samples (KO group). Using Illumina BeadStudio software, we performed a statistical analysis to identify the differentially expressed genes between the *Hdh*-KO and *Hdh*-HET groups. We found that from 45992 probes existing on the Mouse-6 BeadChips arrays, 14,065 probes were present (with detection p-value < 0.01) in at least one of these two groups. Statistical analysis (t-test) has identified 821 transcripts that were significantly different between *Hdh*-HET and *Hdh*-KO groups (p < 0.05) (Fig 6). Among these 821 targets, 455 were up-regulated in the KO group and 366 were down-regulated (Fig 6). Thus, we concluded that inactivation of *Hdh *expression has a very significant effect on the transcriptional profile of MEF cells. The average signal intensities for each probe in *Hdh*-HET and *Hdh*-KO groups are included in the Excel format for the 821 differentially expressed targets (Additional files 1 and 2).

### Analysis and annotation of microarray data

In the annotation of microarray data, we focused on 279 unique genes whose expression differed by at least 2-fold between *Hdh*-HET and *Hdh*-KO groups. From these genes 173 genes (104 of which are annotated) were up-regulated and 106 genes (65 of which were annotated) were down-regulated in *Hdh*-KO cells when compared to *Hdh*-HET cells. In order to extract biological information from these genes, we performed annotation of the array results using the Ingenuity Pathway Analysis platform [36] and the GoStat software [37]. We found that several significant GO categories can be pulled from the data: developmental process (35), nervous system development and function (19), lipid metabolism (18), glucosamine metabolic process (2), transcription regulator activity (5), cellular_component:plasma membrane (16), regulation of cellular process (29), endocytosis (2), mitochondrion (3), extracellular matrix (2), cytoskeleton (5), others (33), indicating the possible disruption of several functional pathways in the absence of Htt (Additional files 1 and 2). We have not observed significant changes in gene expression levels of most proteins involved in Ca^2+ ^signaling pathways (Additional files 1 and 2), indicating that Ca^2+ ^signaling changes observed in our functional experiments (Figs 2, 3, 4) are likely to be due to post-translational effects, such as for example changes in InsP_3_R gating properties.

### Confirmation of gene expression with real-time PCR

**Figure 7:**
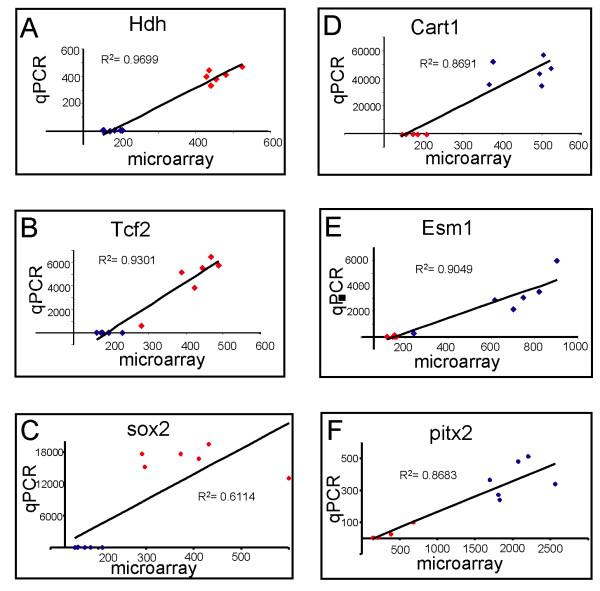
**Correlation between microarray and qPCR results**. The results of the microarray and qPCR analyses are compared for 3 genes downregulated (Hdh, Tcf2, Sox2) and 3 genes upregulated (Cart1, Esm1, Pitx2) in *Hdh*-KO cells. The microarray and qPCR results are shown for RNA extracted from 6 *Hdh*-KO samples (blue) and 6 *Hdh*-HET samples (red).

In order to confirm our microarray results, we performed quantitative real-time PCR (qPCR) analysis for several of the candidate genes. For these experiments we choose three genes that were significantly down-regulated in *Hdh*-KO MEFs (Hdh, Sox-2, Tcf2) and three genes that were significantly up-regulated in *Hdh*-KO MEFs (Cart1, Esm1, Pitx2) (Additional files 1 and 2). We observed a good correlation between averaged microarray results and qPCR data for all six genes evaluated (Table 1). Moreover, we observed a good correlation between microarray and qPCR data for results obtained with cDNA samples from individual *Hdh*-HET and *Hdh*-KO MEF cell lines (Fig 7).

**Table 1:** Comparison of average microarray and qPCR results for 6 selected genes. 3 genes downregulated in Hdh-KO cells are shown in blue, 3 genes upregulated in Hdh-KO cells are shown in red. The average signals obatined in Microarray and qPCR experiments are shown for each gene. Also shown are primers used for qPCR experiments and the fold change calculated from microarry and qPCR data by dividing average *Hdh*-KO signal to average *Hdh*-HET signal.

**Gene**	**GeneBank Acc #**	**Microrray**			**Q-PCR**			
			
		**HET**	**KO**	**Fold**	**Primers (FP/RP)**	**HET**	**KO**	**Fold**
**Hdh**	NM_010414	303.7	-0.9	**DOWN**	CGTGAGCATCTGCCAACATT CACACCGAGGATCAGGAGAGT	404.3	2.0	**-202.2**
**Sox-2**	NM_011443	233.7	-5.6	**DOWN**	GATGCACAACTCGGAGATCAG GCTTCTCGGTCTCGGACAA	16615.9	18.4	**-903.0**
**Tcf2**	NM_009330	255.6	7.3	**-35.0**	CACCTGACAGTAAAATGCAGATCA GAGTGTGTGCTGTGGATATTCGT	4513.7	2.6	**-1736.0**
**Cart1**	NM_172553	-9.3	286.9	**UP**	CCCTGAGAACGGAGCTCACT TTGGCCCTTCGATTTTGAA	12.5	45701.8	**3656.1**
**Esm1**	NM_023612	-35.6	498.6	**UP**	GCGAGGAGGATGATTTTGGT GCATTCCATCCCGAAGGT	19.7	2966.9	**150.6**
**Pitx2**	NM_011098	124.8	1811.8	**14.5**	CCGAGTCCGGGTTTGG TTGCGTTCCCGCTTTCTC	21.3	368.2	**17.3**

### Comparison with gene expression profiling of *Hdh*-null ES cells

When our paper was in preparation, another group independently reported genome-wide expression profiling of Hdh-null embryonic stem (ES) cells [38]. It is of interest to compare our findings with *Hdh*-KO MEF cells with results obtained for Hdh-null ES cells. Strehlow at al (2007) reported that expression of 16 known transcripts was significantly affected in *Hdh-*null ES cells when compared to wild type ES cells. The affected transcripts were divided into several classes of interest: protein degradation (5), extracellular matrix (4), cell division (4), and patterning/development (3) [38]. To compare these data with our results, we attempted to locate the genes highlighted by the study by Strehlow at al (2007) among the genes which expression differs at p < 0.05 between *Hdh*-KO and *Hdh*-HET MEF cells in our experiments. We determined that two of the three genes in the patterning/development category (*Otx2 *and *Pem*) do not present in MEF cell lines, *Leftb/Lefty *is present in MEF cell lines, but did not show a significant difference between HET and KO MEFs. All five genes in the protein degradation category are present in MEF cell lines but did not show significant difference in our experiments. We found that *Adam23 *is not present in MEF cell lines. *B3galt6, col4a1 and clo4a2 *are present in MEF cells, but did not show significant difference. We further found that one out of four genes in cell division category (*Ccdc5*) is also significantly affected in our experiments. *Ccdc5 *is up-regulated in both Hdh-null ES cells and in Hdh-KO MEF cells. *Plk1 *is also strongly upregulated (8.7-fold) in Hdh-null ES cells [38] but did not show significant difference in Hdh-KO MEF cells in our experiments. *Aurkc *is not present in MEF cells, while *Pttg1 *is present but did not show significant difference.

Strehlow at al (2007) induced neuronal differentiation of Hdh-null ES cells by application of retinoic acid and performed microarray analysis of the in vitro differentiated neurons at 6, 8, and 10 days post-differentiation [38]. The expression of transcripts in the number of categories was different between *Hdh*-null and wild type *in vitro *differentiated neurons [38]. Changes in only a few genes have been consistently observed in the two studies (Table 2).

**Table 2:** Comparison of gene expression changes in *Hdh*-KO MEF and differentiated *Hdh*-null ES cells. The genes are classified into functional categories (groups) based on GO classification as explained by Strehlow at al (2007). The number of genes affected in differentiated *Hdh*-null ES cells is shown. The number of these genes present among the genes affected in our experiments with *Hdh*-KO MEF cells is also shown. **The official gene symbols, accession numbers, Diff scores and fold changes are shown for all overlapping genes. The positive Diff score corresponds to the genes upregulated in *Hdh*-KO MEG cells, the negative Diff score corresponds to the genes downregulated in *Hdh*-KO MEF cells. The genes which expression is affected in the opposite direction when *Hdh*-null ES and *Hdh*-KO MEF data are compared are shown in *italic***.

**Gene groups**	**Gene # HMG paper**	**overlap in SupTable 1**	**Overlap gene symbol**	**Accession**	**Diff_Score**	**change fold**
extracellur space	99	1	ilvbl	NM_173751.3	0.037888	2.037322394
extracellular matrix/components conferring tensile strength/cell adhesion	38	2	Emp3 (Ymp)	NM_010129.1	*85.9198*	2.395867383
recepto binding/hormone activity	27	0	Epb4.1l3	NM_013813.1	23.5427	1.63780359
lysosome activity/protein degradation	17	0				
enzyme inhibitor activity	15	0				
growth, proliferation and differentiation	15	1	Emp3 (Ymp)	NM_010129.1	*85.9198*	2.395867383
apoptosis	10	1	Txnl1	NM_016792.2	0.003063	2.178257007
axonogenesis	10	0				
synaptic activity	14	0				
wnt signaling	6	0				
metal ion transport	8	1	Crip1	NM_007763	*0.049374*	5.609568813
transport	4	0				
retinol/retinal/retinoid/isoprenoid binding	5	0				

## Discussion

Despite the importance of understanding the normal function of Htt for both basic biology and for HD, its function remains largely unknown [9,34]. The generation of *Hdh*-null MEF cell lines described in our study provides a new and unbiased approach to search for novel Htt functions.

The Htt directly binds to the InsP_3_R, an intracellular Ca^2+ ^release channel [23,24]. The expression of mutated Htt has been shown to affect the InsP_3_R activity [23] and mitochondrial Ca^2+ ^signals and bioenergetics [25-30]. Thus, in the first series of experiments we evaluated InsP_3_R-mediated Ca^2+ ^cytosolic and mitochondrial Ca^2+ ^signals in *Hdh*-null MEF cells. As a result of these experiments we found that InsP_3_R sensitivity to stimulation by InsP_3 _was reduced in the absence of Hdh (Figs 3 and 4). We further found that Htt appears to be dispensable for ER-mitochondrial Ca^2+ ^coupling (data not shown). Thus the altered InsP_3_R-induced cytoplasmic and mitochondrial calcium signaling in the *Hdh*-null MEF cells may result from the lack of Hdh by itself and does not necessarily require a secondary change in gene regulation.

Interestingly, a large number of Ca^2+^-related genes, such as CACNA2D3 (calcium channel, voltage-dependent, alpha2/delta subunit 3), ITPR1 (inositol 1,4,5-trisphosphate receptor 1), HOMER1 (homer homolog 1), ATP2A2 (ATPase, Ca^2+ ^transporting, cardiac muscle, slow twitch 2), DRD2 (dopamine receptor 2), PRKCB1 (protein kinase C beta 1), PDE1B (phosphodiesterase 1B, Ca^2+^-calmodulin dependent), ATP2B2 (ATPase, Ca^2+ ^transporting, plasma membrane 2), CAMK2B (calcium/calmodulin-dependent protein kinase II, beta), PLCB1 (phospholipase C, beta 1), RGS4 (regulator of G-protein signaling 4), and CAMK2A (calcium/calmodulin-dependent protein kinase II alpha), have been recently reported to be consistently and significantly downregulated in a striatal region of symptomatic human HD patients and aging HD mouse models [39]. These results are in agreement with the "Ca^2+ ^hypothesis of HD" [22] and with a direct role of Htt in intracellular Ca^2+ ^signaling supported by our experiments.

Many studies suggested that Htt also plays a major role in control of gene transcription [33,34]. To search for changes in gene transcription resulting from deletion of Htt gene, we performed a genome-wide comparison of transcription profiles in MEF cells expressing a single copy of *Hdh *(*Hdh*-HET cells) and in MEF cells which lack *Hdh *expression (*Hdh*-KO cells). To minimize sources of variability, the *Hdh*-HET and *Hdh*-KO MEF cells were generated in parallel experiments and on identical genetic background. From our annotation analysis, we found that a large group of affected genes play a role in embryonic development (Additional file 1). This result was not unexpected because Htt is essential for embryonic development, and complete inactivation of Htt expression in knock-out mice causes early embryonic lethality [10-12]. The functions of these genes may provide additional clues about the mechanism responsible for embryonic lethality in *Hdh *knockout mice, for example there are some similar phenotypic manifestations between *Hdh *nullizygous embryos and knockout mutants lacking fibroblast growth factor receptor1(*fgfr1*) [12]. Interestingly, we found *fgfrl1 *message is downregulated approximately 2-fold in *Hdh*-KO MEF cells when compared to *Hdh*-HET cells (Additional files 1 and 2).

After gastrulation, Htt is important for neurogenesis – mice carrying a <50% dose of wild-type Htt display profound malformations of the cortex and striatum [13]. The neuronal inactivation of Htt during mid- to late gestation, for example, leads to neurological abnormalities and progressive degeneration (apoptotic cells in the hippocampus, cortex and striatum, and a lack of axons) [18]. Our analysis revealed a number of genes involved in nervous system development and function which were affected in *Hdh*-KO MEF cell lines (Additional file 1), and this list should also provide useful information to guide further studies of Htt'*s *normal function in the nervous system. For example, *Sox-2 *expression was absent from *Hdh*-KO MEFs. Sox (Sry-related HMG box) genes encode transcription factors regulating crucial developmental decisions in different systems. *Sox2 *is expressed in, and is essential for, totipotent inner cell mass stem cells and other early multipotent cell lineages, and its ablation causes early embryonic lethality [40]. In many different species, *Sox2 *is a marker of the nervous system from the beginning of its development, it maintains a stem-cell like state and actively inhibits neuronal differentiation, S*ox2 *deficiency causes neurodegeneration and impaired neurogenesis in the adult mouse brain. Does the absence of *Sox-2 *play some role in the early embryonic lethality and neurodegeneration in *Hdh *knock-out mice and conditional knock-out mice respectively? Further studies are required to answer these questions. *Sox-11 *is another sox family gene which changes dramatically in *Hdh*-KO MEF cell lines (increases about 10-fold, see Additional files 1 and 2). The widespread expression of *sox-11 *in both the central and peripheral nervous system suggests that *sox-11 *plays a general role in neuronal development, and its changes in *Hdh-*KO cells merit further investigation.

Another group of genes whose expression was significantly affected in *Hdh*-KO MEF cells are the genes related to lipid metabolism. It has been reported in another microarray analysis using clonal striata-derived cells, that genes involved in lipid metabolism were affected after expressing different N-terminal 548-amino-acid Htt fragments [41]. Moreover, recent biochemical data indicated that Htt binds to caveolin and plays a direct role in cholesterol metabolism [42]. All these data suggested that Htt plays an important role in lipid metabolism, which may be affected by HD mutation. Indeed, RXRG (Retinoic acid receptor RXR-gamma) and RBP4 (retinol binding protein 4) are consistently downregulated in a striatal region of symptomatic human HD patients and aging HD mouse models [39].

From our analysis we also found calcium channel voltage-dependent alpha2/delta subunit 1 (Cacna2d1) was down-regulated about 2 fold (Additional files 1 and 2), interestingly the same CACNA2D1 protein has been recently identified as novel Htt-binding partner in unbiased mass-spectroscopy screen [24]. A closely related alpha2/delta subunit 3 (Cacna2d3) was reported on the 3^rd ^place on the list of the genes which are consistently and significantly downregulated in a striatal region of symptomatic human HD patients and aging HD mouse models [39]. As discussed above, these results indicate that Htt may play a role in regulation of Ca^2+ ^channel activity and Ca^2+ ^signaling in cells, consistent with Ca^2+ ^hypothesis of HD [22,43].

## Conclusion

In conclusion, we generated four *Hdh*-HET and four *Hdh*-KO MEF cell lines and performed functional analysis of these cells by Ca^2+ ^imaging methods and genome-wide transcription profiling of these cell lines using a microarray approach. Our results indicated that Htt plays a direct role in intracellular Ca^2+ ^signaling by directly modulating InsP_3_R function in cells. The results of microarray analysis provided a novel and unique information resource for exploring normal function of Htt in cells. The microarray results have been deposited in NCBIs Gene Expression Omnibus (GEO), and are accessible through GEO Series accession number GSE11139 [35]. The *Hdh*-KO cell lines will also serve as a useful tool for future follow-up experiments aimed at elucidating Htt functions *in vivo*.

## Methods

### Generation of *Hdh*-HET and *Hdh*-KO MEF cell lines

Generation and characterization of the *Hdh *+/- and *Hdh*-floxed mice have been described previously [12,18]. E13.5 embryos obtained from a cross between the Hdh-floxed and Hdh +/- mice were first eviscerated and decapitated, and then the carcasses were finely minced using scissors. The tissue obtained from each embryo was digested with 0.25% Trypsin-EDTA at 37°C for 10 min, washed once with 10% FBS in DMEM, and the cell suspensions from each embryo were plated separately in 10% FBS-DMEM culture medium in order to obtain cultures of primary fibroblasts. Following plating of the cells, the genotype of each embryo was determined by PCR [12,18]. After two days in culture, the primary fibroblasts from each genotype (*Hdh *floxed/+ and *Hdh *floxed/-) were pooled together. The pooled cells were then plated on six-well tissue culture plates, grown to 60–80% confluence and transfected with SV40-Large T-antigen plasmid in pcDNA3-Zeo vector (linearized with PvuI) using the Fugene-6 lipofection reagent (Roche). Transfected cells were cultured for four-six weeks until immortalized *Hdh *floxed/+ and *Hdh *floxed/- mouse embryonic fibroblasts (MEFs) were obtained. *Hdh *floxed/+ and Hdh floxed/- immortalized MEFs were then infected with Lenti-NLS-GFP-Cre virus [19] encoding a nuclear-targeted GFP-Cre fusion protein. Expression of NLS-GFP-Cre in infected cells catalyzed recombination at the *Hdh*(flox) loxP sites leading to excision of the promoter and exon 1 sequences of *Hdh*(flox) allele [18]. Following infection with Lenti-NLS-GFP-Cre, the *Hdh *floxed/+ and *Hdh *floxed/- MEFs were plated in 10% FBS-DMEM culture medium in 96 well plates at an average density of one cell/well for clonal selection. Successful recombination with NLS-GFP-Cre converts the *Hdh *floxed/+ MEFs to *Hdh *+/- (*Hdh*-HET) MEFs and the *Hdh *floxed/- MEFs to *Hdh*-/-(*Hdh*-KO) MEFs. After four weeks in culture, several potential *Hdh*-HET and *Hdh*-KO MEF lines were selected, expanded and analyzed by Western blotting with monoclonal antibodies against Hdh (Chemicon MAB2166, 1:500) and monoclonal antibodies against actin (Chemicon MAB1501, 1:2000).

### Cytosolic and mitochondrial Ca^2+ ^imaging

For measurements of mitochondrial matrix [Ca^2+^] ([Ca^2+^]_m_), the cells were transfected with a mitochondrial matrix targeted inverse pericam construct [44] by electroporation 48–72 h prior to the imaging experiment. Before use, the cells were preincubated in an extracellular medium (2% BSA/ECM) consisting of 121 mM NaCl, 5 mM NaHCO_3_, 10 mM Na-HEPES, 4.7 mM KCl, 1.2 mM KH_2_PO_4_, 1.2 mM MgSO_4_, 2 mM CaCl_2_, 10 mM glucose and 2% bovine serum albumin (BSA), pH 7.4. To monitor [Ca^2+^]_c _cells were loaded with 2.5 μM Fura2/AM for 20–30 min in the presence of 200 μM sulfinpyrazone and 0.003% (w/v) pluronic acid at room temperature. Before start of the measurement the buffer was replaced by a Ca^2+ ^free 0.25%BSA/ECM ([Ca^2+^] <1 μM).

Coverslips were mounted on the thermo stated stage (35°C) of an Olympus IX70 inverted microscope fitted with a 40× (UApo, NA 1.35) oil immersion objective. Fluorescence images were collected using a cooled CCD camera (Pluto, Pixelvision). Excitation was rapidly switched among 340 and 380 nm for fura2 and 495 nm for pericam and a 510 nm longpass dichroic mirror and a 520 nm longpass emission filter were used.

For evaluation of cytoplasmic [Ca^2+^] ([Ca^2+^]_c_), Fura2 fluorescence was calculated for the total area of individual cells. [Ca^2+^]_c _was calibrated in terms of nM using in vitro dye calibration. For evaluation of [Ca^2+^]_m _the pericam-mt signal was masked. Recordings obtained from all transfected cells on the field (2–10 cells) were averaged for comparison in each experiment. Experiments were carried out with at least four different cell preparations. Significance of differences from the relevant controls was calculated by ANOVA.

### Measurements of cytosolic Ca^2+ ^in permeabilized cells

The cells (2 mg protein/1.5 ml) were permeabilized in an intracellular medium (KCl 120 mM, NaCl 10 mM, KH_2_PO_4 _1 mM, Tris-HEPES 20 mM, and antipain, leupeptin and pepstatin 1 μg/ml each at pH 7.2) supplemented with 40 μg/ml digitonin and with fura2/FA (1.5–3 μM) or furaFF/FA (0.5 μM) and TMRE 2 μM in a stirred thermo stated cuvette at 35°C. All the measurements were carried out in the presence of succinate 2 mM, 2 mM MgATP and ATP regenerating system composed of 5 mM phosphocreatine, 5 U/ml creatine kinase. Fura2/FA or FuraFF/FA was added to monitor [Ca^2+^] in the intracellular medium that exchanges readily with the cytosolic space and so [Ca^2+^]_fura2 _was abbreviated as [Ca^2+^]_c_. Fluorescence was monitored in a fluorometer (Delta-RAM, PTI) using 340 nm, 380 nm excitation and 500 nm emission for fura2FF and 540 nm excitation and 580 nm emission for TMRE. Calibration of the fura fluorescence was carried out at the end of each measurement as described previously [45]. Experiments were with at least five different cell preparations in multiple parallels. Significance of differences from the relevant controls was calculated by ANOVA.

### Microarray analysis

Total RNA was isolated from fibroblast cultures using the TRIZOL reagent according to manufacturer's instructions (Invitrogen). Briefly, the MEF cells were grown to 60–80% confluence in T25 tissue culture flask, the culture medium was aspired and 1 ml of TRIZOL reagent was added to each flask. The cells were incubated with TRIZOL at room temperature for 5 min. The resulting lysates were collected from each flask, mixed with 0.2 ml of chloroform and centrifuged at 12,000 × g for 15 min at 4°C. The supernatants were collected, mixed with an equal volume of 70% ethanol at room temperature and immediately transferred to RNAeasy mini spin columns for RNA purification according to the manufacturer's (Qiagen) instructions. The final RNA samples were eluted from the RNeasy mini spin columns in 30 μl of DEPC-treated water. Using the procedures described above, we isolated total RNA from *Hdh*-HET MEF lines 1, 2 (in duplicate), 3, and 5 (in duplicate), and from *Hdh*-KO MEF lines 11, 12, 16 (in duplicate), 27 (in duplicate). The resulting 12 samples were submitted to the UT Southwestern Microarray Core Facility for microarray analysis. Biotinylated cRNA was prepared using the Illumina RNA Amplification Kit (Ambion, Inc., Austin, TX) according to the manufacturer's directions starting with ~200 ng total RNA. Samples were purified using the RNeasy kit (Qiagen, Valencia, CA). Hybridization to the Sentrix Mouse-6 Expression BeadChip (Illumina, Inc., San Diego, CA), washing and scanning were performed according to the Illumina BeadStation 500× manual (revision C). Two BeadChips were used, each one containing 6 arrays. For each chip, three HET and three KO samples were analyzed to minimize the effects of chip-to-chip variability. Arrays were scanned with an Illumina Bead array Reader confocal scanner and the data was analyzed using Illumina's BeadStudio software(Version 3). The raw data was background subtracted and normalized using "cubic spline" method in the software. The detection p values were computed using a dynamically constructed normal model based on the intensities of 700 negative controls. For differential analysis, the six arrays in HET group were compared with the six arrays in KO group using t-test with computing false discovery rate algorithm. The genes with p value <0.05 were considered differentially expressed and subject for further analysis.

### Quantitative Real Time PCR

Quantitative real time (qRT)-PCR was performed using an Applied Biosystems Prism 7900HT sequence detection system using SYBR green chemistry. Briefly, total RNA was treated with DNase I (RNase-free, Roche Molecular Biochemicals), and reverse-transcribed with random hexamers using SuperScript II reverse transcriptase (Invitrogen) to generate cDNA as previously described [46]. Primers were designed using Primer Express Software (PerkinElmer Life Sciences) and validated by analysis of template titration and dissociation curves. Each qRT-PCR contained (final volume of 10 μl) 25 ng of reverse-transcribed RNA, each primer at 150 nM, and 5 μl of 2× SYBR Green PCR Master Mix (Applied Biosystems), and each sample was analyzed in triplicate. Results were evaluated by the comparative C_T _method (User Bulletin No. 2, PerkinElmer Life Sciences) using cyclophilin as the invariant control gene.

## Authors' contributions

HZ generated the Hdh-KO and Hdh-HET cell lines, carried out microarray experiments and prepared results for publication, SD and GH performed Ca^2+ ^imaging studies and prepared results for publication, QZL assisted with microarray experiments and analysis and prepared results for publication, ID and SZ generated critical reagents and help with interpretation of obtained results, JJR assisted with qPCR experiments and analysis, IB conceived the study, and participated in its design and coordination and helped to draft the manuscript. All authors read and approved the final manuscript.

## Supplementary Material

Additional file 1 **Supplementary Table 1. The list of annotated genes significantly affected in *Hdh*-KO MEF cells**. The genes are classified into significant GO categories (classes) as explained in the text. The official gene symbol and protein name are shown. Also shown is average intensity of microarray signal for *Hdh*-HET and *Hdh*-KO samples and the calculated Diff score and p-values. Negative Diff scores correspond to genes underexpressed in *Hdh*-KO cells, positive Diff Scores correspond to genes overexpressed in *Hdh*-KO cells. The fold change is calculated by dividing average *Hdh*-HET and *Hdh*-KO signals. The transcripts which are not detected in *Hdh*-HET samples are shown as "up", the samples not detected in *Hdh*-KO samples are shown as "down". The Genebank accession numbers are also shown. Click here for file

Additional file 2 **Excell file with Supplementary Table 1**. The microarray data for 821 differentially expressed probes are presented in the Excell file. The data listed for each probe are as follows: an Illumina beads target ID, an average signal from 6 chips with *Hdh*-HET samples, the probability of detection of a probe in *Hdh*-HET samples, an average signal from 6 chips with *Hdh*-KO samples, the probability of detection of a probe in *Hdh*-KO samples, the Diff Score obtained by comparing *Hdh*-HET and *Hdh*-HET samples, the P value calculated from the Diff score, an official gene symbol and definition (when available), the sequence of the probe, and the genebank accession number (when available). The results are sorted by the Diff score with negative Diff scores corresponding to the probes downregulated in Hdh-KO cells (366 probes) and positive Diff scores corresponding to the probes upregulated in Hdh-KO cells (455 probes). Click here for file
