# Supplementary material for: Elucidating a normal function of huntingtin by functional and microarray analysis of huntingtin-null mouse embryonic fibroblasts
Source: BMC Neurosci. 2008 Apr 15;9:38. doi: 10.1186/1471-2202-9-38 (PMC2377268; doi:10.1186/1471-2202-9-38)
Supplement: Additional file 1 — Supplementary Table 1. The list of annotated genes significantly affected in Hdh-KO MEF cells. The genes are classified into significant GO categories (classes) as explained in the text. The official gene symbol and protein name are shown. Also shown is average intensity of microarray signal for Hdh-HET and Hdh-KO samples and the calculated Diff score and p-values. Negative Diff scores correspond to genes underexpressed in Hdh-KO cells, positive Diff Scores correspond to genes overexpressed in Hdh-KO cells. The fold change is calculated by dividing average Hdh-HET and Hdh-KO signals. The transcripts which are not detected in Hdh-HET samples are shown as "up", the samples not detected in Hdh-KO samples are shown as "down". The Genebank accession numbers are also shown. [file 1471-2202-9-38-S1.doc]

**Table 1. The list of annotated genes significantly affected in Hdh-KO MEF cells.**

The genes are classified into significant GO categories (classes) as explained in the text. The official gene symbol and protein name are shown. Also shown is average intensity of microarray signal for *Hdh*-HET and *Hdh*-KO samples and the calculated Diff score and p-values. Negative Diff scores correspond to genes underexpressed in *Hdh*-KO cells, positive Diff Scores correspond to genes overexpressed in *Hdh*-KO cells. The fold change is calculated by dividing average *Hdh*-HET and *Hdh*-KO signals. The transcripts which are not detected in *Hdh*-HET samples are shown as “up”, the samples not detected in *Hdh*-KO samples are shown as “down”. The Genebank accession numbers are also shown. The results within each category are sorted by the Diff score.

| **GO category/class** | **SYMBOL** | gene name | ***Hdh*-HET** | ***Hdh*-KO** | **change fold** | **Diff Score** | **Pval** | **ACCESSION** |
| --- | --- | --- | --- | --- | --- | --- | --- | --- |
| embryonic development | Hdh | huntingtin | 302.0929 | -2.304878 | down | -31.83252 | 0.0006558 | NM_010414 |
| (developmental process) | Hoxa7 | homeo box A7 | 700.6027 | 77.58759 | 9.0298294 | -24.54399 | 0.0035124 | NM_010455 |
|  | Card10 | caspase recruitment domain family member 10 | 610.6219 | 179.1202 | 3.4090064 | -21.21375 | 0.0075618 | NM_130859 |
|  | Mid2 | midline 2 | 243.1566 | 46.09888 | 5.274674786 | -20.20463 | 0.0095397 | AK085856 |
|  | Kirrel3 | kin of IRRE like 3 | 402.9307 | 91.9245 | 4.3832787 | -19.50366 | 0.0112107 | NM_026324 |
|  | Tcf2 | transcription factor 2, hepatic | 253.8828 | 6.002152 | 42.298629 | -18.56569 | 0.0139133 | NM_009330 |
|  | Crebbp | CREB binding protein | 612.8356 | 269.0348 | 2.2779046 | -15.5525 | 0.0278452 | XM_148699 |
|  | Nsd1 | nuclear receptor-binding SET-domain protein 1 | 102.5286 | 36.43489 | 2.8140225 | -14.50457 | 0.035444 | NM_008739 |
|  | Fgfrl1 | fibroblast growth factor receptor-like 1 | 321.1121 | 159.818 | 2.0092361 | -13.10683 | 0.048901 | NM_054071 |
|  | Sox5 | SRY-box containing gene 5 | 115.5461 | 18.31506 | 6.3088027 | -13.04627 | 0.0495876 | NM_011444 |
|  | Rhob | ras homolog gene family member B | 2230.813 | 6513.396 | 2.9197409 | 13.30392 | 0.0467313 | NM_007483 |
|  | Esm1 | endothelial cell-specific molecule 1 | -36.93542 | 497.195 | up | 13.35501 | 0.0461848 | NM_023612 |
|  | Vcam1 | vascular cell adhesion molecule 1 | 1982.301 | 5760.005 | 2.9057166 | 13.81217 | 0.0415702 | NM_011693 |
|  | Igfbp6 | insulin-like growth factor binding protein 6 | 72.51485 | 623.8224 | 8.6026848 | 14.19871 | 0.0380303 | NM_008344 |
|  | Itga6 | integrin alpha 6 | 518.332 | 1810.98 | 3.4938611 | 14.21928 | 0.0378505 | NM_008397 |
|  | Lck | lymphocyte protein tyrosine kinase | 66.09452 | 187.8988 | 2.8428802 | 14.24778 | 0.037603 | NM_010693 |
|  | Prrx2 | paired related homeobox 2 | 363.5406 | 1023.898 | 2.8164612 | 14.49087 | 0.035556 | XM_149096 |
|  | Dact1 | dapper homolog 1 antagonist of beta-catenin | 0.659129 | 620.1953 | 940.93159 | 16.59177 | 0.0219191 | NM_021532 |
|  | Eomes | eomesodermin homolog | 1.672702 | 144.8513 | 86.597194 | 16.68703 | 0.0214436 | NM_010136 |
|  | Emp3 | epithelial membrane protein 3 | 855.1215 | 2048.706 | 2.3958069 | 17.36898 | 0.0183274 | NM_010129 |
|  | Bin1 | bridging integrator 1 | 119.3868 | 317.3471 | 2.6581423 | 17.43393 | 0.0180554 | NM_009668 |
|  | Snx2 | sorting nexin 2 | 129.601 | 313.4163 | 2.418317 | 17.60096 | 0.0173742 | NM_026386 |
|  | Tcof1 | Treacher Collins Franceschetti syndrome 1 homolog | 741.1921 | 1697.726 | 2.2905344 | 17.92121 | 0.0161391 | NM_011552 |
|  | Ripk3 | receptor-interacting serine-threonine kinase 3 | 781.8918 | 2220.036 | 2.8393136 | 18.3741 | 0.0145408 | NM_019955 |
|  | Hspa2 | heat shock protein 2 | 1522.458 | 3285.343 | 2.1579203 | 18.56569 | 0.0139133 | NM_008301 |
|  | Nme5 | expressed in non-metastatic cells 5 | 90.71925 | 265.3258 | 2.9246913 | 18.74717 | 0.0133439 | NM_080637 |
|  | Myo7a | myosin VIIa | 33.75101 | 106.362 | 3.1513724 | 19.31369 | 0.011712 | NM_008663 |
|  | Hsf4 | heat shock transcription factor 4 | -0.3004668 | 90.05511 | up | 19.86237 | 0.010322 | NM_011939 |
|  | Bmp1 | bone morphogenetic protein 1 | 390.4132 | 842.2898 | 2.1574317 | 19.86237 | 0.010322 | NM_009755 |
|  | Meox1 | mesenchyme homeobox 1 | 12.85038 | 206.5502 | 16.07347 | 20.97277 | 0.0079932 | NM_010791 |
|  | Crabp1 | cellular retinoic acid binding protein I | 194.6768 | 3244.903 | 16.668155 | 21.15285 | 0.0076686 | NM_013496 |
|  | Txnl1 | thioredoxin-like 1 | 1236.967 | 2695.316 | 2.1789716 | 25.13852 | 0.003063 | NM_016792 |
|  | Pitx2 | paired-like homeodomain transcription factor 2 | 123.1848 | 1809.288 | 14.687591 | 29.991 | 0.0010021 | NM_011098 |
|  | Eif4ebp3 | eukaryotic translation initiation factor 4E binding protein 3 | 251.9981 | 678.0593 | 2.6907318 | 30.1346 | 0.0009695 | NM_201256 |
|  | Cobl | cordon-bleu | 25.17485 | 159.9357 | 6.3529952 | 33.02369 | 0.0004985 | NM_172496 |
|  |  |  |  |  |  |  |  |  |
| nervous system development and function | Rdh10 | retinol dehydrogenase 10 (all-trans) | 983.4281 | 282.5991 | 3.479940665 | -24.78621 | 0.0033218 | NM_133832 |
|  | Nab1 | Ngfi-A binding protein 1 | 100.4848 | 1.657597 | 60.620766 | -21.69098 | 0.0067749 | NM_008667 |
|  | Ctsc | cathepsin C | 709.927 | 207.0079 | 3.429468151 | -17.43393 | 0.0180554 | NM_009982 |
|  | Elovl4 | elongation of very long chain fatty acids (FEN1/Elo2 SUR4/Elo3 yeast)-like 4 | 907.5578 | 368.8351 | 2.460605837 | -16.01779 | 0.0250162 | NM_148941 |
|  | Sox2 | SRY-box containing gene 2 | 231.7812 | -6.957195 | down | -15.35846 | 0.0291175 | NM_011443 |
|  | Ngef | neuronal guanine nucleotide exchange factor | 285.582 | 97.17764 | 2.9387625 | -14.40049 | 0.0363037 | NM_019867 |
|  | Fem1c | fem-1 homolog c (C.elegans) | 42.34457 | 114.0901 | 2.694326569 | 13.89876 | 0.0407496 | NM_175117 |
|  | Ctf1 | cardiotrophin 1 | 5.003942 | 132.7837 | 26.535819 | 14.24794 | 0.0376016 | NM_007795 |
|  | Cln2 | ceroid-lipofuscinosis neuronal 2 | 807.8345 | 1781.462 | 2.205231393 | 14.63785 | 0.0343728 | NM_009906 |
|  | Ccnd2 | cyclin D2 | 57.03332 | 263.6131 | 4.6220893 | 14.78978 | 0.0331911 | AK077367 |
|  | Slit3 | slit homolog 3 | 19.1822 | 176.4915 | 9.2007955 | 15.5097 | 0.028121 | XM_203363 |
|  | Utrn | utrophin | 10.57514 | 94.21652 | 8.9092456 | 17.24351 | 0.0188647 | NM_011682 |
|  | Shox2 | short stature homeobox 2 | 306.6682 | 4009.108 | 13.073113 | 18.10563 | 0.0154681 | NM_013665 |
|  | Mbp | myelin basic protein | 17.73081 | 149.7483 | 8.4456548 | 20.01484 | 0.0099659 | AK039047 |
|  | Cdk5rap1 | CDK5 regulatory subunit associated protein 1 | 48.61112 | 154.5043 | 3.1783736 | 21.70887 | 0.006747 | NM_025876 |
|  | Esr2 | estrogen receptor 2 (beta) | 5.378811 | 91.80384 | 17.067683 | 21.83763 | 0.0065499 | NM_010157 |
|  | Cart1 | cartilage homeo protein 1 | -10.71455 | 285.6816 | up | 22.21234 | 0.0060085 | NM_172553 |
|  | Tagln3 | transgelin 3 | 9.79881 | 110.1779 | 11.244008 | 26.88955 | 0.0020467 | NM_019754 |
|  | Sox11 | SRY-box containing gene 11 | 252.5385 | 2506.545 | 9.9253975 | 28.35411 | 0.0014608 | NM_009234 |
|  |  |  |  |  |  |  |  |  |
| lipid metabolism | Scarb1 | scavenger receptor class B member 1 | 4188.733 | 2027.227 | 2.0662378 | -22.20569 | 0.0060177 | NM_016741 |
|  | Sec14l2 | SEC14-like 2 | 595.4863 | 278.0089 | 2.1419685 | -19.02678 | 0.0125119 | NM_144520 |
|  | Plscr2 | phospholipid scramblase 2 | 1935.823 | 428.6451 | 4.516144 | -18.32213 | 0.0147159 | NM_008880 |
|  | Hdlbp | high density lipoprotein binding protein | 555.5875 | 270.4695 | 2.0541595 | -16.63674 | 0.0216933 | NM_133808 |
|  | Pik3c2a | phosphatidylinositol 3-kinase C2 domain containing alpha polypeptide | 153.1353 | 57.75066 | 2.6516632 | -15.73801 | 0.0266808 | NM_011083 |
|  | Etnk1 | ethanolamine kinase 1 | 137.4287 | 67.75405 | 2.0283466 | -14.24778 | 0.037603 | XM_284250 |
|  | Sytl4 | synaptotagmin-like 4 | 371.1741 | 39.77402 | 9.3320741 | -13.7955 | 0.0417302 | NM_013757 |
|  | Ncoa1 | nuclear receptor coactivator 1 | 113.6483 | 30.17361 | 3.76648 | -13.74544 | 0.042214 | NM_010881 |
|  | Acsl4 | acyl-CoA synthetase long-chain family member 4 | 385.5797 | 180.0601 | 2.1413945 | -13.08129 | 0.0491893 | NM_207625 |
|  | Zfyve1 | zinc finger FYVE domain containing 1 | 88.50253 | 183.0522 | 2.0683273 | 13.88832 | 0.0408477 | NM_183154 |
|  | Tm7sf2 | transmembrane 7 superfamily member 2 | 122.8187 | 247.8183 | 2.0177571 | 14.36227 | 0.0366246 | XM_129087 |
|  | Fabp3 | fatty acid binding protein 3 | 1484.145 | 4762.692 | 3.2090476 | 14.87051 | 0.0325798 | NM_010174 |
|  | Lpl | lipoprotein lipase | 27.3145 | 528.1874 | 19.337253 | 15.66586 | 0.0271278 | NM_008509 |
|  | Grb10 | growth factor receptor bound protein 10 | 26.65586 | 101.9304 | 3.8239397 | 18.56569 | 0.0139133 | NM_010345 |
|  | Pik3r4 | phosphatidylinositol 3 kinase regulatory subunit polypeptide 4 p150 | 296.1845 | 635.5983 | 2.145953958 | 19.30784 | 0.0117278 | XM_356182 |
|  | Plekha2 | pleckstrin homology domain-containing family A member 2 | 1218.553 | 3078.103 | 2.5260313 | 21.46507 | 0.0071366 | NM_031257 |
|  | Atp9b | ATPas class II type 9B | 283.5472 | 739.6628 | 2.6086056 | 29.991 | 0.0010021 | NM_015805 |
|  | Renbp | renin binding protein | 114.588 | 2690.899 | 23.483253 | 33.02369 | 0.0004985 | NM_023132 |
|  |  |  |  |  |  |  |  |  |
| glucosamine metabolic process | Pgm3 | phosphoglucomutase 3 | 91.00799 | 36.04126 | 2.5251057 | -14.02721 | 0.039562 | NM_028352 |
|  | Gnpda1 | glucosamine-6-phosphate deaminase 1 | 41.4902 | 388.2801 | 9.3583569 | 18.32213 | 0.0147159 | XM_124695 |
|  |  |  |  |  |  |  |  |  |
| transcription regulator activity | Tcfcp2l1 | Transcription factor CP2-like1 | 2074.964 | 36.5334 | 56.796356 | -15.5313 | 0.0279815 | NM_023755 |
|  | Foxd1 | forkhead box D1 | 2263.752 | 107.6921 | 21.020595 | -14.81012 | 0.0330361 | NM_008242 |
|  | Klf2 | Kruppel-like factor 2 | 449.5738 | 1367.447 | 3.0416519 | 14.24093 | 0.0376623 | NM_008452 |
|  | Mbd1 | methyl-CpG binding domain protein 1 | 293.0075 | 649.4645 | 2.2165457 | 15.45023 | 0.0285087 | NM_013594 |
|  | Apbb3 | amyloid beta (A4) precursor protein-binding family B member 3 | 134.7535 | 291.2346 | 2.161239597 | 26.57002 | 0.0022029 | NM_146085 |
|  |  |  |  |  |  |  |  |  |
| cellular_component:plasma membrane | Tmem24 | transmembrane protein 24 | 1191.478 | 414.5229 | 2.874335773 | -27.89437 | 0.0016239 | XM_134795 |
|  | Itm2a | integral membrane protein 2A | 322.9799 | 97.95292 | 3.297297314 | -25.186 | 0.0030297 | NM_008409 |
|  | Loxl4 | lysyl oxidase-like 4 | 2754.768 | 1181.936 | 2.330725183 | -19.92479 | 0.0101747 | AK014773 |
|  | Ppp1r3f | protein phosphatase 1 regulatory (inhibitor) subunit 3F | 279.2614 | 98.297 | 2.840996165 | -18.10563 | 0.0154681 | NM_138605 |
|  | Slc6a6 | solute carrier family 6 member 6 | 202.8187 | 75.02518 | 2.7033417 | -15.19424 | 0.0302396 | AK041141 |
|  | Hig1 | hypoxia induced gene 1 | 400.694 | 178.5885 | 2.243671905 | -15.14162 | 0.0306082 | NM_019814 |
|  | Ifngr2 | interferon gamma receptor 2 | 282.6859 | 131.7846 | 2.1450602 | -14.40049 | 0.0363037 | NM_008338 |
|  | Slc6a17 | Solute carrier family 6 member 17 | 163.1463 | 39.57283 | 4.1226847 | -14.12095 | 0.0387173 | NM_172271 |
|  | Csf2ra | colony stimulating factor 2 receptor alpha low-affinity | 181.4885 | 43.80618 | 4.1429885 | -14.04224 | 0.0394254 | NM_009970 |
|  | Cacna2d1 | calcium channel voltage-dependent alpha2/delta subunit 1 | 441.0106 | 220.0092 | 2.0045098 | -13.89876 | 0.0407496 | NM_009784 |
|  | Siglec10 | sialic acid binding Ig-like lectin 10 | 73.3987 | 335.7158 | 4.57386575 | 13.79426 | 0.0417421 | NM_172900 |
|  | Fut11 | fucosyltransferase 11 | 106.3343 | 239.4914 | 2.252249744 | 14.04224 | 0.0394254 | XM_203633 |
|  | Il17rd | interleukin 17 receptor D | 48.25172 | 116.1897 | 2.407990845 | 14.28313 | 0.0372981 | NM_134437 |
|  | Cnn2 | calponin 2 | 4998.226 | 10855.56 | 2.1718826 | 17.92121 | 0.0161391 | NM_007725 |
|  | Raet1e | retinoic acid early transcript 1E | 26.94126 | 105.1412 | 3.9026089 | 18.355 | 0.0146049 | NM_198193 |
|  | Aqp5 | aquaporin 5 | 16.2895 | 253.4072 | 15.556475 | 21.30157 | 0.0074104 | NM_009701 |
|  |  |  |  |  |  |  |  |  |
| regulation of cellular process | Gnb4 | guanine nucleotide binding protein beta 4 | 990.7952 | 346.4521 | 2.859833149 | -34.23761 | 0.0003769 | NM_013531 |
|  | Pank1 | pantothenate kinase 1 | 175.4177 | 77.11697 | 2.274696477 | -26.17432 | 0.0024131 | NM_023792 |
|  | Arl6ip5 | ADP-ribosylation factor-like 6 interacting protein 5 | 1638.915 | 816.6677 | 2.006832155 | -19.02678 | 0.0125119 | NM_022992 |
|  | Dlgh3 | discs large homolog 3 (Drosophila) | 1150.909 | 568.3824 | 2.024885007 | -18.99553 | 0.0126022 | NM_016747 |
|  | Lactb | lactamase beta | 355.7363 | 172.2449 | 2.06529366 | -17.92121 | 0.0161391 | NM_030717 |
|  | Sash1 | SAM and SH3 domain-containing protein 1 | 1054.079 | 425.8884 | 2.4750122 | -16.26849 | 0.023613 | NM_175155 |
|  | Mrpl36 | mitochondrial ribosomal protein L36 | 1157.736 | 460.1672 | 2.515902915 | -15.93113 | 0.0255204 | NM_053163 |
|  | Sfmbt2 | Scm-like with four mbt domains 2 | 87.04398 | 0.6325176 | 137.61511 | -14.93175 | 0.0321237 | NM_177386 |
|  | Nrk | Nik related kinase | 130.2255 | 15.61477 | 8.3398923 | -14.79959 | 0.0331162 | NM_013724 |
|  | Cgref1 | cell growth regulator with EF hand domain 1 | 131.1983 | 18.86028 | 6.9563283 | -14.53744 | 0.0351768 | XM_181420 |
|  | Hspbap1 | Hspb associated protein 1 | 1328.673 | 635.6146 | 2.090375205 | -14.49196 | 0.0355471 | NM_175111 |
|  | Grit | Rho GTPase-activating protein | 161.103 | 62.37438 | 2.5828393 | -14.44853 | 0.0359043 | NM_177379 |
|  | Tpd52l1 | tumor protein D52-like 1 | 217.351 | 64.47324 | 3.371181594 | -14.34011 | 0.036812 | NM_009413 |
|  | Rasl12 | RAS-like, family 12 | 322.025 | 121.7618 | 2.644712874 | -13.95503 | 0.0402251 | XM_134870 |
|  | Erdr1 | erythroid differentiation regulator 1 | 2451.731 | 1224.089 | 2.002902567 | -13.24628 | 0.0473556 | NM_133362 |
|  | Crip1 | Mus musculus cysteine-rich protein 1 (intestinal) (Crip1) mRNA. | 167.777 | 948.4109 | 5.652806404 | 13.06505 | 0.0493736 | NM_007763 |
|  | Diras1 | DIRAS family GTP-binding RAS-like 1 | 43.57399 | 201.172 | 4.616790888 | 13.53152 | 0.0443454 | NM_145217 |
|  | Ilvbl | ilvB (bacterial acetolactate synthase)-like | 1081.817 | 2204.01 | 2.037322394 | 14.21497 | 0.0378881 | NM_173751 |
|  | Ptpn2 | protein tyrosine phosphatase non-receptor type 2 | 135.0646 | 285.3397 | 2.112616481 | 14.59653 | 0.0347014 | AK076072 |
|  | Dtx3 | deltex 3 homolog (Drosophila) | 1055.458 | 2149.758 | 2.036801085 | 15.06928 | 0.0311224 | NM_030714 |
|  | Nars | asparaginyl-tRNA synthetase | 3547.943 | 7474.368 | 2.10667646 | 15.86965 | 0.0258842 | NM_027350 |
|  | Lars | leucyl-tRNA synthetase | 6520.938 | 13821.64 | 2.119578502 | 18.22335 | 0.0150545 |  |
|  | Lgals9 | lectin galactose binding soluble 9 | 478.1717 | 1775.786 | 3.7136995 | 18.74717 | 0.0133439 | NM_010708 |
|  | Rad51l1 | RAD51-like 1 (S. cerevisiae) | 136.9328 | 280.5032 | 2.048473412 | 18.74717 | 0.0133439 | NM_009014 |
|  | Ascc1 | activating signal cointegrator 1 complex subunit 1 | 501.1915 | 1158.415 | 2.3113221 | 19.30784 | 0.0117278 | XM_483908 |
|  | Mcts1 | malignant T cell amplified sequence 1 | 630.9933 | 1384.047 | 2.193441674 | 20.73856 | 0.0084361 | NM_026902 |
|  | Fech | ferrochelatase | 364.5677 | 847.985 | 2.326001453 | 21.83763 | 0.0065499 | NM_007998 |
|  | Statip1 | signal transducer and activator of transcription interacting protein 1 | 1749.795 | 3748.854 | 2.1424533 | 22.21234 | 0.0060085 | NM_021448 |
|  | Riok3 | RIO kinase 3 (yeast) | 1325.561 | 2920.448 | 2.20317888 | 25.54026 | 0.0027924 | NM_024182 |
|  |  |  |  |  |  |  |  |  |
| endocytosis | Mrc2 | mannose receptor C type 2 | 44.86067 | 107.3447 | 2.392846562 | 15.88375 | 0.0258003 | AK054150 |
|  | Fchsd1 | FCH and double SH3 domains 1 | 326.7064 | 683.6212 | 2.092463447 | 22.77703 | 0.0052759 | NM_175684 |
|  |  |  |  |  |  |  |  |  |
| mitochondrion | Immp2l | inner mitochondrial membrane peptidase 2-like (S. cerevisiae) | 112.3339 | 257.2306 | 2.289875096 | 13.98223 | 0.039974 | NM_053122 |
|  | Nme4 | expressed in non-metastatic cells 4 protein | 805.3594 | 1969.705 | 2.445746582 | 14.56586 | 0.0349473 | NM_019731 |
|  | Papd1 | PAP associated domain containing 1 | 535.1516 | 1090.084 | 2.036962984 | 22.20569 | 0.0060177 | NM_026157 |
|  |  |  |  |  |  |  |  |  |
| extracellular matrix | Adamts10 | a disintegrin-like and metalloprotease (reprolysin type) with thrombospondin type 1 motif 10 | 36.59117 | 89.88197 | 2.45638415 | 17.92121 | 0.0161391 | NM_172619 |
|  | Tsrc1 | thrombospondin repeat containing 1 | 835.2347 | 2010.5 | 2.407107846 | 19.56851 | 0.0110446 | NM_144899 |
|  |  |  |  |  |  |  |  |  |
| cytoskeleton | Tuba4 | tubulin alpha 4 | 269.377 | 87.91732 | 3.063981022 | -15.7892 | 0.0263682 | NM_009447 |
|  | Krt1-18 | keratin complex 1 acidic gene 18 | 29.51852 | 98.23392 | 3.327874162 | 13.21924 | 0.0476514 | NM_010664 |
|  | Rai14 | retinoic acid induced 14 | 2601.399 | 6125.396 | 2.354654553 | 13.6952 | 0.0427051 | NM_030690 |
|  | Pdlim2 | PDZ and LIM domain 2 | 72.41712 | 195.0984 | 2.694092226 | 19.1433 | 0.0121806 | NM_145978 |
|  | Ccdc5 | coiled-coil domain containing 5 | 1757.315 | 3583.341 | 2.039099991 | 26.35444 | 0.002315 | NM_146089 |
|  |  |  |  |  |  |  |  |  |
| others | Gm440 | Rap2 binding protein 9 | 194.6157 | 16.21497 | 12.00222387 | -33.02369 | 0.0004985 | NM_198620 |
|  | Zfp537 | zinc finger protein 537 | 253.45 | 12.96568 | 19.54775993 | -22.21234 | 0.0060085 | NM_172298 |
|  | Trrp1 | transient receptor protein 1 | 175.9107 | 45.90908 | 3.831719128 | -18.47433 | 0.0142091 | AK005144 |
|  | Zfp288 | zinc finger protein 288 | 393.061 | 151.9308 | 2.587105445 | -18.34546 | 0.0146371 | XM_147215 |
|  | Mela | melanoma antigen | 392.0762 | 21.16532 | 18.5244636 | -17.17686 | 0.0191564 | NM_008581 |
|  | Glcci1 | glucocorticoid induced transcript 1 (Glcci1) | 281.4704 | 77.81231 | 3.617299114 | -16.29812 | 0.0234525 | NM_178072 |
|  | Nudel-pending | nuclear distribution gene E-like | 263.7582 | 119.3222 | 2.210470474 | -15.49891 | 0.0281909 | AK011168 |
|  | Cpeb3 | cytoplasmic polyadenylation element binding protein 3 | 123.4927 | 27.43725 | 4.500913904 | -14.50457 | 0.035444 | NM_198300 |
|  | Rgpr | regucalcin gene promotor region related protein | 124.7908 | 40.89022 | 3.051849562 | -14.36227 | 0.0366246 | NM_033354 |
|  | Ccl25 | chemokine (C-C motif) ligand 25 | 99.28512 | 34.93858 | 2.8417045 | -13.53152 | 0.0443454 | NM_009138 |
|  | Hspa12a | heat shock 70kDa protein 12A | 115.3585 | 32.89838 | 3.506510047 | -13.53152 | 0.0443454 | NM_175199 |
|  | G6pc3 | glucose 6 phosphatase catalytic 3 | 115.5469 | 274.6338 | 2.376816687 | 13.04627 | 0.0495876 | NM_175935 |
|  | Ttc7b | TPR repeat protein 7b | 220.7259 | 604.9352 | 2.740662514 | 13.25728 | 0.0472359 | XM_127105 |
|  | Oplah | 5-oxoprolinase (ATP-hydrolysing) | 75.40302 | 197.0647 | 2.613485508 | 13.27545 | 0.0470387 | NM_153122 |
|  | Ddc8 | testis specific protein Ddc8 | 166.2359 | 498.8316 | 3.000745326 | 13.65823 | 0.0430702 | NM_021440 |
|  | Ttc15 | TPR repeat protein 15 | 65.75312 | 192.1199 | 2.921837017 | 13.7689 | 0.0419865 | NM_178811 |
|  | Zfp521 | zinc finger protein 521 | 21.09966 | 145.2352 | 6.88329575 | 14.04224 | 0.0394254 | NM_145492 |
|  | Zfp35 | zinc finger protein 35 | 155.7864 | 316.2825 | 2.030231779 | 14.24778 | 0.037603 | NM_011755 |
|  | Dos | downstream of Stk11 (Dos) mRNA. | 234.283 | 764.78 | 3.264342697 | 14.81012 | 0.0330361 | XM_125771 |
|  | Zfh4 | zinc finger homeodomain 4 (Zfh4) mRNA. | 145.336 | 325.564 | 2.240078164 | 15.11871 | 0.0307701 | NM_030708 |
|  | Lace1 | lactation elevated 1 | 67.96938 | 158.4739 | 2.331548412 | 15.12849 | 0.0307009 | NM_145743 |
|  | Mppe1 | metallophosphoesterase 1 | 313.407 | 641.2859 | 2.046176059 | 16.49178 | 0.0224296 | NM_172630 |
|  | Cirbp | cold inducible RNA binding protein | 206.9038 | 511.9012 | 2.474102457 | 18.34546 | 0.0146371 | NM_007705 |
|  | Glb1l | Galactosidase beta-1-like protein | 56.11553 | 189.2672 | 3.372813194 | 18.74717 | 0.0133439 |  |
|  | Zfp41 | zinc finger protein 41 | 368.5984 | 746.9377 | 2.026426865 | 19.06775 | 0.0123944 | NM_011759 |
|  | Gsdmdc1 | Gasdermin domain-containing protein 1 | 8.419005 | 120.2211 | 14.27972783 | 19.07753 | 0.0123665 | NM_026960 |
|  | Wbscr17 | Williams-Beuren syndrome chromosome region 17 homolog | 104.6659 | 611.7177 | 5.844479434 | 20.91082 | 0.0081081 | NM_145218 |
|  | Zfp397 | zinc finger protein 397 | 79.96859 | 209.744 | 2.622829789 | 20.97277 | 0.0079932 | NM_027007 |
|  | Lrrc15 | leucine rich repeat containing 15 | 48.12303 | 789.0388 | 16.39628261 | 22.21234 | 0.0060085 | XM_358823 |
|  | Atp6v1g2 | ATPase H+ transporting V1 subunit G isoform 2 | 72.81575 | 452.0738 | 6.208461768 | 23.3551 | 0.0046184 | NM_023179 |
|  | Wac | WW domain containing adaptor with coiled-coil | 788.0099 | 1638.886 | 2.079778439 | 25.43354 | 0.0028618 | NM_153085 |
|  | Hspa12b | heat shock protein 12B | 47.09647 | 263.5912 | 5.596835601 | 29.53683 | 0.0011125 | NM_028306 |
|  | Wdr7 | WD repeat domain 7 | 85.73422 | 269.0514 | 3.138203159 | 29.54093 | 0.0011115 | XM_140391 |
